# Influence of osmolality on gastrointestinal fluid volume and drug absorption: potential impact on oral salt supplementation

**DOI:** 10.1186/s40780-021-00212-z

**Published:** 2021-09-01

**Authors:** Miyuki Takemura, Yuki Tanaka, Katsuhisa Inoue, Ikumi Tamai, Yoshiyuki Shirasaka

**Affiliations:** 1 Faculty of Pharmacy, Institute of Medical, Pharmaceutical and Health Sciences, Kanazawa University, Kakuma-machi, Kanazawa, 920-1192 Japan; 2 School of Pharmacy, Tokyo University of Pharmacy and Life Sciences, Hachioji, Tokyo, Japan

**Keywords:** Drug absorption, Gastrointestinal tract, Pharmacokinetics, Osmolality, Membrane permeability

## Abstract

**Background:**

The syndrome of inappropriate secretion of antidiuretic hormone (SIADH) is the most frequent cause of hyponatremia in patients with cerebrovascular disease, and is often treated with oral salt tablets. However, we have shown that osmolality-dependent variations in gastrointestinal (GI) fluid volume can alter the concentration of a poorly permeable drug in the GI tract, potentially affecting its absorption. Here, we examined the effect of ingestion of hyperosmotic solution (10% NaCl) on drug concentration and absorption in the GI tract.

**Methods:**

The effects of osmolality on luminal fluid volume and drug absorption in rat intestine (jejunum, ileum and colon) were examined by means of an in situ closed loop method using fluorescein isothiocyanate-dextran 4000 (FD-4) and atenolol. In vivo absorption in rats was determined by measuring the plasma concentration after oral administration of the test compounds dissolved in purified water or hyperosmotic solution (10% NaCl).

**Results:**

Administration of hyperosmotic solution directly into the GI tract significantly increased the GI fluid volume, owing to secretion of water into the lumen. After administration in hyperosmotic solution, the luminal concentration of non-permeable FD-4 was significantly lower than the initial dosing concentration, whereas after administration in purified water, the luminal concentration exceeded the initial concentration. The fraction absorbed of atenolol was markedly lower after administration in hyperosmotic solution than after administration in purified water. An in vivo pharmacokinetic study in rats was consistent with these findings.

**Conclusions:**

Administration of hyperosmotic NaCl solution increased GI fluid volume and reduced the plasma level of orally administered atenolol. This may imply that oral salt tablets used to treat hyponatremia in SIADH patients could decrease the intestinal absorption of concomitantly administered drugs, resulting in lower plasma exposure.

## Introduction

The gastrointestinal (GI) concentration of an orally administered drug depends upon the luminal fluid volume, and in turn, the drug concentration influences the absorption kinetics. Indeed, we recently showed that osmolality-dependent variations in GI fluid volume can cause significant changes of drug concentration in the GI tract [1]. Specifically, the fraction absorbed of atenolol (a low-permeability drug) was significantly greater when administered in purified water than when administered in isosmotic solution, while there was no significant difference in the fraction absorbed of antipyrine (a high-permeability drug). We also showed that the high-osmolality environment in the GI tract resulting from oral ingestion of apple juice induces secretion of water into the lumen, resulting in a reduction of luminal concentration and decreased absorption of coadministered atenolol [2].

Thus, changes in the osmotic environment or fluid volume in the GI tract might have clinically relevant effects. For example, enteral nutrition is theoretically isosmotic, but may result in decreased absorption of drugs [1]. Furthermore, salt supplementation in patients with hyponatremia may make the GI tract more hyperosmotic. For example, the syndrome of inappropriate secretion of antidiuretic hormone (SIADH) is the most frequent cause of hyponatremia in patients with cerebrovascular disease, and causes a volume-expanded state due to antidiuretic hormone-mediated renal fluid retention, resulting in a dilution of serum sodium. The first-line therapy for SIADH is fluid restriction [3]. Treatment often involves intravenous 3% NaCl or oral administration of salt tablets or 10% NaCl, a hyperosmotic solution [4]. Therefore, SIADH patients are likely to have a high-osmolality environment in the GI tract, and this is expected to influence the luminal fluid volume.

In the present study, we carried out in situ and in vivo experiments using fluorescein isothiocyanate-dextran 4000 (FD-4, a non-permeable compound) and atenolol (a low-permeability drug) in order to examine whether ingestion of hyperosmotic solution (10% NaCl) alters the intestinal absorption of atenolol by changing the drug concentration in the GI tract.

## Methods

### Materials

FD-4 was purchased from Sigma-Aldrich Company (St. Louis, Missouri, USA). Atenolol and NaCl were purchased from Wako Pure Chemical Industries, Ltd. (Osaka, Japan). All other compounds and reagents were obtained from Nacalai Tesque, Inc. (Kyoto, Japan), Wako Pure Chemical Industries, Ltd., or Sigma-Aldrich Company.

### Animals

Male Wistar rats were purchased from Tokyo Laboratory Animals Science Co., Ltd. (Tokyo, Japan) and NINOX Labo Supply Inc. (Ishikawa, Japan). All animal experimental protocols were reviewed and approved by the Committee of Animal Care and Welfare of Tokyo University of Pharmacy and Life Sciences and Kanazawa University. Male Wistar rats were housed three per cage with free access to commercial chow and tap water, and were maintained on a 12 h dark/light cycle (08:00–20:00 light) in an air-controlled room (temperature, 24.0 ± 1 °C; humidity, 55 ± 5%).

### Measurement of osmolality of experimental solutions

Test compounds (FD-4 or atenolol) were dissolved in purified water or hyperosmotic solution (10% NaCl). For osmolality measurements of experimental solutions, hyperosmotic solution (10% NaCl) was diluted with purified water. The supernatant was collected after filtration, and the osmolality was measured with a cryoscopic osmometer, OSMOMAT 030-D (Gonotec GmbH, Berlin, Germany).

### In situ intestinal closed loop experiment

The in situ rat intestinal closed loop method was carried out as described previously [1, 5]. Male Wistar rats (7–8 weeks old; 250 ± 20 g body weight) were fasted overnight and anesthetized by intraperitoneal injection of a triple anesthetic combination (medetomidine, midazolam and butorphanol). The abdominal cavity was opened and an intestinal loop (jejunum, 10 cm; ileum, 10 cm; colon, 7 cm) was made by cannulation into both ends of the jejunum, ileum and colon with silicone tubing (i.d., 3 mm). The intestinal contents were washed out through the cannulas with saline. Test compounds (FD-4 or atenolol) were dissolved in purified water or hyperosmotic solution (10% NaCl). One mL of test compound solution (10 μM) was introduced into the intestinal loop and both ends of the loop were ligated. At the designated times (30 min), test compound solution in the loop was collected by flushing with air (for measuring the luminal concentration of test compound, *C*_out_), and then made up to 10 mL with buffer solution (for measuring the amount of test compound, *X*_out_). The volume of luminal water (mL) in each segment of intestine was calculated using the following equation:

1 $$ {V}_{\mathrm{water}}=\frac{X_{\mathrm{out}}}{C_{\mathrm{out}}} $$

where *V*_water_ (mL) is the water volume in each segment of intestine, *C*_out_ is the luminal concentration of test compound (μM), and *X*_out_ is the amount of test compound (μmol). The fraction absorbed of test compound was calculated from the remaining amount of test compound in each intestinal loop. Final results were normalized to the surface area calculated from the radius and length of each segment of intestine. The radii of the small and large intestines reported by Fagerholm et al. were used (0.178 cm and 0.252 cm, respectively) [6].

### In vivo pharmacokinetic study in rats

An in vivo pharmacokinetic study in rats was carried out as described previously [2, 7–9]. Male Wistar rats (7–8 weeks old; 250 ± 20 g body weight) were fasted overnight and anesthetized by intraperitoneal injection of a triple anaesthetic combination (medetomidine, midazolam and butorphanol). Prior to administration of atenolol, the right jugular vein was cannulated with silicone tubing (100-00 N; 0.5 mm I.D., 1.0 mm O.D., Kaneka Medical Products). Groups of six rats were orally administered atenolol (1 mg/kg, 0.25 mg/mL) solution in purified water or hyperosmotic solution (10% NaCl) by gavage. The rats could move freely and were not anesthetized during the experiment. Blood samples (500 μL) were collected from the cannula into heparinized tubes at designated times (up to 420 min). Each blood sample was replaced with an equal volume of saline and heparinized saline was used to maintain the patency of the catheter. Blood samples were centrifuged at 900×g for 10 min. The resultant plasma was added to 5 times volume of acetonitrile containing propranolol (internal standard) and the reactions were incubated for 60 min. Protein was then removed by centrifugation and supernatants were stored at − 30 °C until analysis.

Plasma concentration-time curves of atenolol were plotted and analyzed. The pharmacokinetics of atenolol after oral administration were estimated by means of non-compartmental analysis using the MOMENT program [10]. The AUC values of atenolol from 0 to 7 h (AUC_0–7_) and from 0 to infinity (AUC_0–∞_) were calculated using the linear trapezoidal rule. The maximum plasma concentration (*C*_max_) and the time to reach maximum plasma concentration (*t*_max_) were obtained directly from the experimental data. The apparent elimination half-life of the log-linear phase (*t*_1/2_) was calculated based on the terminal elimination rate constant determined by log-linear regression of the final data points (at least 3).

### Analytical methods

Concentrations of FD-4 were measured with a microplate fluorescence reader (VarioskanTM Flash 2.4; Thermo Fisher Scientific Inc., Kanagawa, Japan) at excitation/emission wavelengths of 492/515 nm. Atenolol in all samples was quantified with a liquid chromatography-tandem mass spectrometry (LC-MS/MS) system.

Atenolol was quantified with a LC-MS/MS system consisting of an MDS-Sciex API 3200™ triple quadrupole mass spectrometer (AB SCIEX, Foster City, CA) coupled with a LC-20 AD ultra-fast LC (UFLC) system (Shimadzu Company, Kyoto, Japan). An Agilent Eclitse plus (C_18_, 50 × 2.1 mm, 5 μm) was used as the analytical column. Gradient elution was performed with a mobile phase composed of 0.1% formic acid (A) and acetonitrile (B) at a flow rate of 0.4 mL/min. The gradient profile for atenolol was 2% B for 0–1.5 min, 2.0–80% B for 1.5–2.0 min, 80% B for 2.0–4.5 min, 80–50% B for 4.5–5.0 min, 50% B for 5.0–6.0 min, 50–2.0% B for 6.0–6.5 min, and 2% B for 6.5–7.0 min. The total run time was 7.0 min. The mass transitions (Q1/Q3) of m/z 267.2/145.2 and 260.1/116.2 were used for atenolol and propranolol (internal standard), respectively. Analyst software version 1.4.2 (AB SCIEX) was used for data manipulation.

### Statistical analysis

Data are given as the mean of values obtained in at least three experiments with the standard error (SEM). The statistical significance of differences for two-group was evaluated using the unpaired Student’s t-test. A probability of less than 0.05 (*p* < 0.05) was considered to be statistically significant.

## Results

### Influence of osmolality of orally administered solutions on remaining fraction of water and luminal concentration of FD-4 in rats

**Fig. 1:**
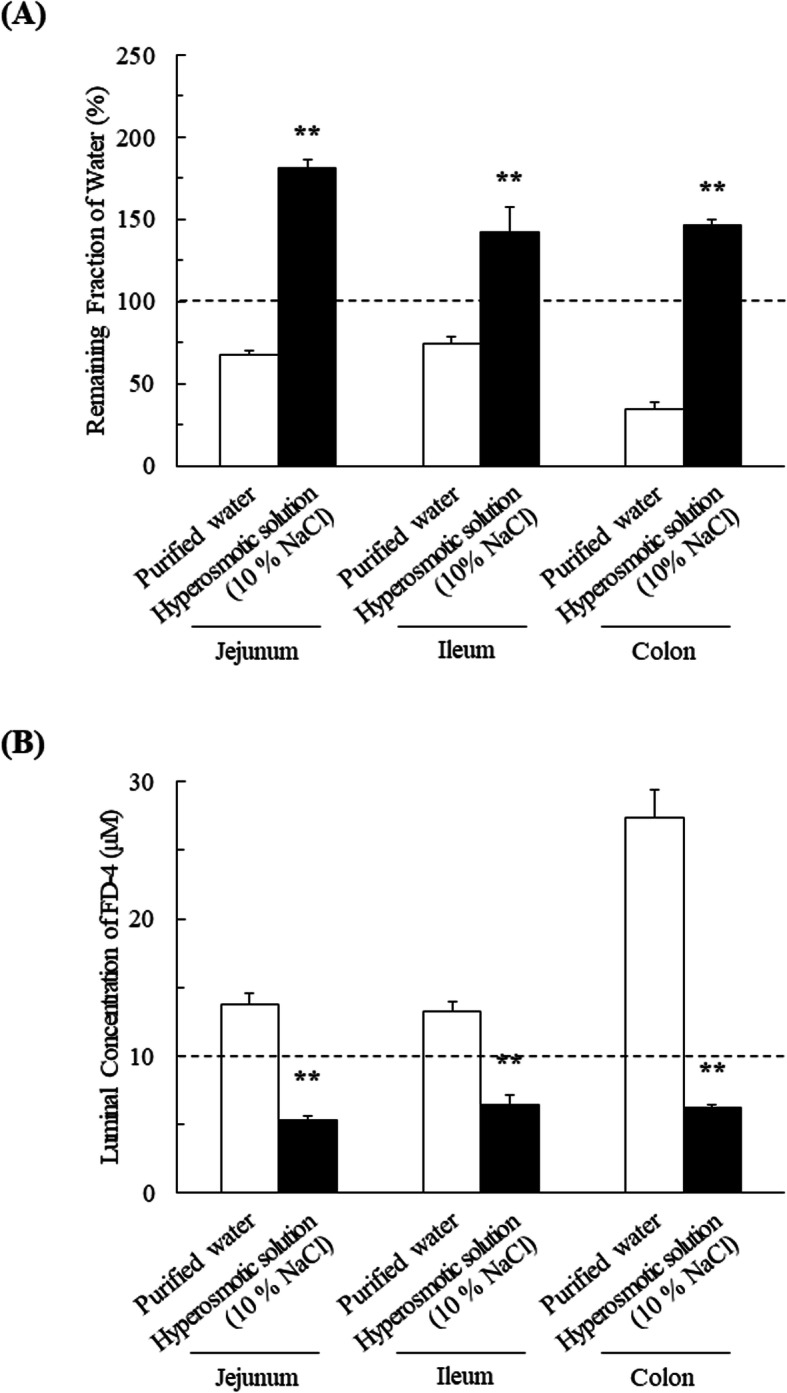
Influence of osmolality of orally administered solutions on remaining fraction of water and luminal concentration of FD-4 in rats. **A** The remaining fraction of water and (**B**) luminal concentration of FD-4 in jejunum, ileum and colon after administration of FD-4 (10 μM) in purified water and hyperosmotic solution (10% NaCl) were measured by means of an in situ closed loop method for 30 min at 37 °C. The statistical significance between the different conditions was evaluated using an unpaired Student t-test. *P < 0.05, **P < 0.01, significantly different from purified water. Data are shown as means ± SEM (*n* = 6–9)

To investigate the effect of the osmolality of the administered solution on GI fluid absorption, the fluid volumes in jejunum, ileum, and colon were examined after the ingestion of purified water and hyperosmotic solution (10% NaCl) (Fig.1A). The values of remaining fraction of water at 30 min after the ingestion of purified water were 67.9 ± 2.0% (jejunum), 74.4 ± 3.8% (ileum), and 34.6 ± 3.7% (colon), while the corresponding values after the ingestion of hyperosmotic solution were 181 ± 4% (jejunum), 142 ± 15% (ileum), and 146 ± 3% (colon), respectively. The remaining fraction of water was significantly higher in the case of hyperosmotic solution, as compared with purified water, in all segments. The osmolality of hyperosmotic solution used in this study was measured to be 3412 ± 5 mOsm/kg. Therefore, it is reasonable to consider that water secretion into the lumen may be occurred due to the high-osmolality condition in the GI tract.

The luminal concentration of FD-4, a non-permeable compound, was measured in jejunum, ileum, and colon after ingestion with purified water and hyperosmotic solution (Fig. 1B). Luminal concentrations of FD-4 at 30 min after administration in purified water were 13.7 ± 0.8 (jejunum), 13.2 ± 0.7 (ileum), and 27.4 ± 2.0 μM (colon), whereas those after administration in hyperosmotic solution were 5.30 ± 0.31(jejunum), 6.47 ± 0.68 (ileum), and 6.25 ± 0.15 μM (colon). Thus, the intestinal concentration of FD-4 after administration in hyperosmotic solution was much smaller than that in purified water in all segments.

### Effect of osmolality of orally administered solutions on luminal concentration and fraction absorbed of atenolol in rats

**Fig. 2:**
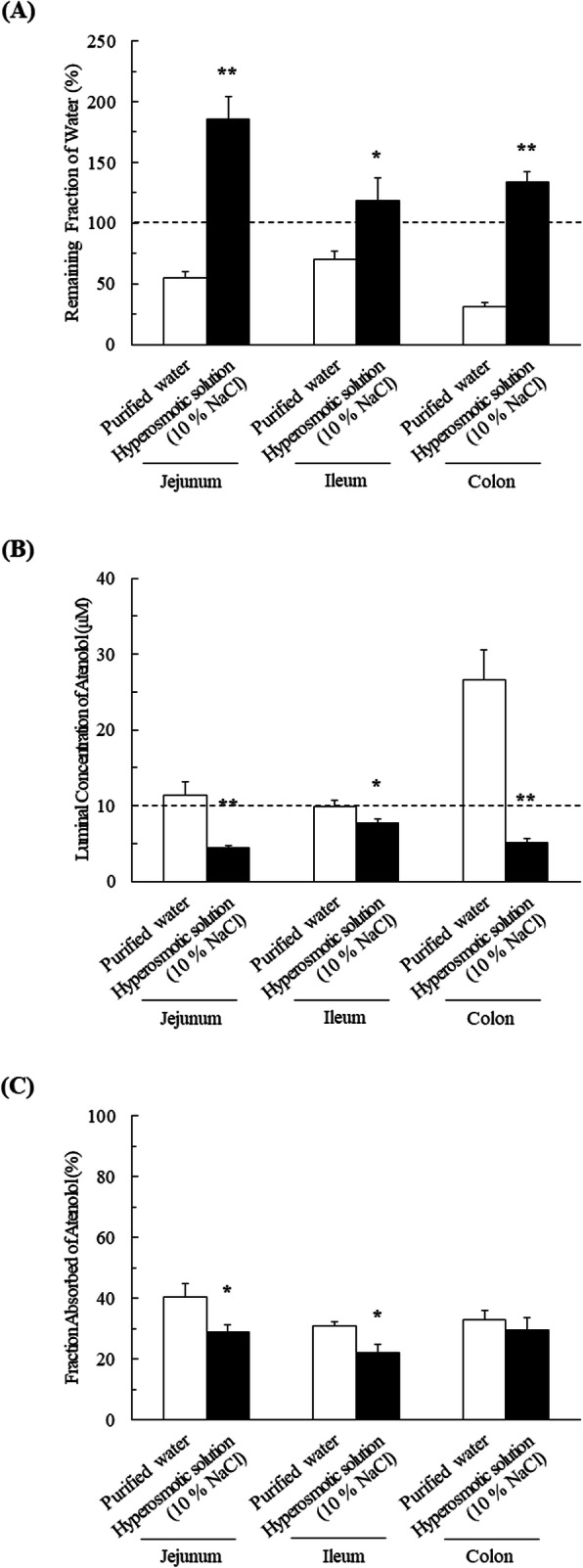
Impact of osmolality of orally administered solutions on luminal concentration and fraction absorbed of atenolol in rats. **A** The remaining fraction of water, (**B**) luminal concentration of atenolol and (**C**) fraction absorbed of atenolol in jejunum, ileum and colon after administration of atenolol (10 μM) in purified water and hyperosmotic solution (10% NaCl) were determined by means of the in situ closed loop method for 30 min at 37 °C. The statistical significance between the different conditions was evaluated using an unpaired Student t-test. *P < 0.05, **P < 0.01, significantly different from purified water. Data are shown as means ± SEM (*n* = 6–9)

To examine the effect of solution osmolality on intestinal absorption of atenolol, the luminal concentration and fraction absorbed of atenolol in jejunum, ileum, and colon were determined after administration in purified water and hyperosmotic solution (10% NaCl) (Fig. 2). As shown in Fig. 2A, the values of remaining fraction of water at 30 min after administration of atenolol in purified water were 54.8% ± 5.1% (jejunum), 70.3% ± 6.6% (ileum), and 30.8% ± 3.5% (colon), whereas the corresponding values after administration in hyperosmotic solution were 185% ± 18% (jejunum), 118% ± 19% (ileum), and 134% ± 8% (colon). This is consistent with the results shown in Fig. 1A.

As shown in Fig. 2B, the luminal concentrations of atenolol at 30 min after administration in purified water were 11.4 ± 1.7 (jejunum), 9.93 ± 0.73 (ileum), and 26.5 ± 4.0 μM (colon), while those after administration in hyperosmotic solution were 4.40 ± 0.32 (jejunum), 7.66 ± 0.63 (ileum), and 5.08 ± 0.53 μM (colon). Meanwhile, the values of fraction absorbed of atenolol at 30 min after administration in purified water were 40.4% ± 4.2% (jejunum), 30.8% ± 1.2% (ileum), and 32.7% ± 3.4% (colon), while those after administration in hyperosmotic solution were 29.0 ± 2.1 (jejunum), 22.2 ± 2.7 (ileum), and 29.5 ± 3.9 μM (colon) (Fig. 2C). These results suggest that the absorption of atenolol in jejunum and ileum is significantly less after administration in hyperosmotic solution than after administration in purified water.

### Impact of osmolality of orally administered solutions on atenolol absorption in rats

When atenolol (1 mg/kg) was orally administered with 1 ml purified water to rats, the area under the plasma concentration-time curve from 0 to 7 h (AUC_0–7_) and the maximum plasma drug concentration (*C*_max_) of atenolol were 212 ± 11 ng·h/mL and 68.4 ± 4.7 ng/ml, respectively. Coadministration of hyperosmotic solution (10% NaCl) with atenolol significantly decreased the AUC_0–7_ and *C*_max_ to 42.0 and 54.9%, respectively. On the other hand, purified water and hyperosmotic solution did not significantly alter the time to reach maximum plasma concentration (*t*_max_) or the apparent elimination half-life (*t*_1/2_) of atenolol (Table 1).

**Table 1:** Pharmacokinetic parameters of atenolol after oral administration in rats

Pharmacokinetic parameters ^*a*^		Purified Water	Hyperosmotic Solution (10% NaCl)
		(*n* = 6)	(*n* = 6)
AUC_0–7_	(ng·h/mL)	212 ± 11	89.0 ± 25.4^**^
AUC_0-∞_	(ng·h/mL)	231 ± 11	92.9 ± 27.6^**^
*C*_max_	(ng/mL)	68.4 ± 4.7	37.6 ± 9.2^*^
*t*_max_	(h)	2.30 ± 0.30	1.92 ± 0.08
*t*_1/2_	(h)	1.59 ± 0.15	1.38 ± 0.41

Atenolol (1 mg/kg, 0.25 mg/mL) was orally administered with purified water or hyperosmotic solution (10% NaCl)

^*a*^*AUC* area under plasma concentration-time curve; *C*_*max*_ maximum plasma concentration; *t*_*max*_ time to reach maximum plasma concentration; *t*_*1/2*_ elimination half-life

The statistical significance between the different conditions was evaluated using an unpaired Student t-test. *P < 0.05, **P < 0.01, significantly different from control values. Data are shown as means ± S.E. (*n* = 6)

## Discussion

Cerebrovascular disease patients have a high risk of hyponatremia and associated complications. Hyponatremia in these patients is usually either due to SIADH or cerebral salt wasting syndrome (CSWS). The common treatment for these diseases is increased sodium intake, and especially in SIADH, restriction of fluid intake is also necessary because of fluid retention. The recommended treatment for SIADH is to limit the total daily fluid intake to 15–20 mL/kg of body weight, and to administer at least 200 mEq of salt per day [11]. Clinical samples have treatment with intravenous 3% NaCl or oral administration of salt tablets or 10% NaCl, a hyperosmotic solution [4]. Consequently, the GI tract may be exposed to a high-osmolality environment during treatment. Our present results show that administration of hyperosmotic solution increased the GI fluid volume (Fig. 1A). This would have been due to secretion of water into the lumen, because the osmolality of the administered hyperosmotic solution was measured to be 3412 ± 5 mOsm/kg, resulting in a high-osmolality condition in the GI tract. Interestingly, in previous studies, no significant difference of GI fluid movement was observed between saline (0.9% NaCl) and purified water [1, 2]. This was explained in terms of water absorption after administration of saline due to a decrease of osmolality as a result of Na^+^ and/or Cl^−^ absorption. Indeed, NaCl absorption in the intestinal epithelium participates in the coordinated action of the SLC9/Slc9 family of Na^+^/H^+^ exchangers (e.g. NHE2, NHE3) and the SLC26/Slc26 family of Cl^−^/HCO_3_^−^ exchangers (e.g. SLC26A3/Slc26a3, SLC26A6/Slc26a6) in both rats and humans [12–16]. Therefore, our present results imply that in the case of hyperosmotic solution (10% NaCl), the osmolality-dependent water transfer occurs more rapidly than NaCl absorption (Fig. 1A). In this context, it was reported that NaCl overload decreases the intestinal expression of NHE3 (Slc9a3) and the electroneutral Na^+^/HCO_3_^−^ cotransporter NBCn2 (Slc4a10) in rats. In other words, the down-regulation of NBCn2 induced by high NaCl intake may reduce the absorption capacity of NaCl from the small intestine [14]. Consequently, administration of hyperosmotic NaCl solution might maintain a high-osmolality environment in the GI tract for a prolonged period, resulting in significant water transfer, compared to administration of purified water.

**Fig. 3:**
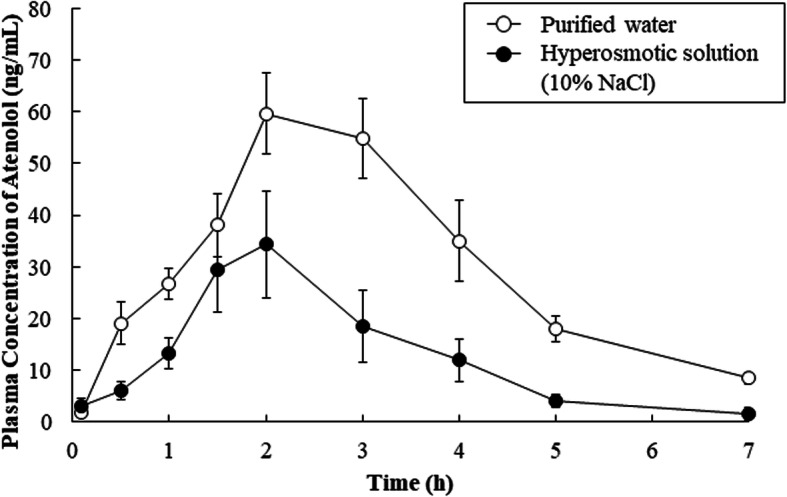
Mean plasma concentration-time profiles of atenolol in rats after oral administration. Mean plasma concentration-time profiles of atenolol in rats after oral administration. Atenolol (1 mg/kg, 0.25 mg/ml) was orally administered with purified water (open circles) or hyperosmotic solution (closed circles). Data are shown as the means ± S.E. (*n* = 6)

After administration in hyperosmotic solution, the luminal concentration of non-permeable FD-4 was significantly lower than the initial dosing concentration, while the luminal concentration after administration in purified water exceeded the initial concentration (Fig. 1B). These results suggest that osmolality-dependent variations in GI fluid volume due to ingested solutions may influence absorption of a drug by altering its concentration in the GI tract. Indeed, the luminal concentration and fraction absorbed of atenolol were markedly lower after administration in hyperosmotic solution than after administration in purified water (Figs. 2B and C). This can be explained by fluid secretion after ingestion of the hyperosmotic solution, thereby reducing the drug concentration, and leading to decreased absorption due to the lower concentration gradient. These considerations are supported by our in vivo observation that the plasma concentration of atenolol following oral administration with hyperosmotic solution was significantly reduced (Fig. 3 and Table 1).

In Fig. 1A and Fig. 2A, the luminal fluid volume after intestinal administration of hyperosmotic solution (1.0 mL) increased to approximately 1.8 mL (corresponding to approximately 180% remaining fraction). Assuming that similar effect was observed in the human GI tract, ingested fluid volume (hyperosmotic solution (15–20 mL/kg/day)) may apparently increase 1.8-fold, potentially resulting in a decrease of coadministered drug concentration, which in turn leads to decreased absorption [4, 11]. However, this effect has potential to be attenuated by existence of a steady fluid volume in the GI tract. Indeed, recent report showed that a steady volume around 77 mL (range of 15–172 mL) was observed in the human GI tract [17]. Meanwhile, the steady fluid volume in the GI tract of rats is also reported to be approximately 3 mL [18]. Therefore, we speculate that our in vivo results shown in Fig. 3 can reflect the phenomenon observed in human in vivo.

Our previous study suggested that osmolality-dependent variations in GI fluid volume may indirectly influence intestinal absorption, especially for drugs with low permeability (Biopharmaceutics Classification System (BCS) Class III and IV), but not high permeability (BCS Class I and II) [1]. The β-blocker atenolol, employed as a model drug in this study, is a low-permeability drug used to treat hypertension, a risk factor for cerebrovascular disease. Thus, patients with cerebrovascular disease may be taking it when they have complications of hyponatremia. In clinical practice, furosemide, a loop diuretic, and tolvaptan, a selective antagonist of vasopressin V2 receptors, are recommended for the treatment of hyponatremia [19]. Because both of furosemide and tolvaptan are low-permeability drugs (BCS Class IV), it is possible that oral salt tablets may alter the pharmacokinetics of these drugs in hyponatremic patients who need to restrict fluid intake.

## Conclusion

We found that GI fluid volume is strongly influenced by the administration of hyperosmotic NaCl solution, and this causes reduced intestinal absorption of atenolol by decreasing the drug concentration in the GI tract. Our findings suggest that administration of oral salt tablets to SIADH patients might result in decreased plasma exposure to concomitant orally administered drugs.

## Data Availability

All data generated or analyzed during this study are included in this published article.

## Abbreviations

GI: Gastrointestinal
FD-4: FITC-dextran
SIADH: Syndrome of inappropriate secretion of antidiuretic hormone
CSWS: Cerebral salt wasting syndrome
BCS: Biopharmaceutics classification system
